# Why Studies in the Effect of Positive Psychological Interventions Should Use Life-Satisfaction as an Outcome

**DOI:** 10.3389/fpsyg.2021.758623

**Published:** 2021-11-25

**Authors:** Ruut Veenhoven

**Affiliations:** ^1^Erasmus Happiness Economics Research Organization, Erasmus University Rotterdam, Rotterdam, Netherlands; ^2^Optentia Research Program, Noth-West University, Vanderbijlpark, South Africa

**Keywords:** positive mental health, positive psychological interventions, hedonic happiness, outcome measures, measurement of positive mental health

## Abstract

The effect of positive psychological interventions (PPIs) is mostly assessed using self-report measures of positive mental health. These measures are problematic because (1) the content addressed is often not clear, (2) different scales are used to assess different notions of positive mental health, which impedes comparability, (3) the concept of positive mental health involves objective capabilities which are not well measurable using subjective self-estimates, and (4) the concept behind the measures denotes presumed chances for adaptation to life rather than adaptation as such. Therefore, we should also measure the effect of PPIs using life-satisfaction, which is (a) a clear-cut concept and as such tells us what an intervention brings about, (b) is well measurable using self-reports, since it is a subjective concept, (c) it allows better comparability across studies, and (d) it indicates actual adaptation to life instead of strengthening of presumed of chances for adaptation.

## Introduction

It is widely agreed that a stronger evidence base is required to support claims about the effectiveness of positive psychological interventions (PPIs), a promise made at the interception of the movement in the late 1990s (Seligman and Csikszentmihalyi, 2000). This is needed to deal with current levels of skepticism around the value of PPIs, and the resulting underuse of these techniques. To demonstration that PPIs have any effect, we must assess how large any effects of PPIs are and what type works best for whom. Fortunately, there is a growing stream of studies on the effects of PPIs on which recent meta-analyses have been performed, e.g., by Bolier et al. (2013), Chakhssi et al. (2018), and White et al. (2019). However, this strand of effect research has met with several problems, which, in my view, requires us to shift our focus from positive mental health (eudaimonic happiness) to life-satisfaction (hedonic happiness) as an outcome variable. Here, I discuss the problems of using measures of positive mental health to evaluate PPIs and argue why we should also use life-satisfaction as an outcome variable of PPIs.

## Different Notions of Positive Mental Health

The first problem lies in the notion of positive mental health itself; this is a fuzzy concept that denotes a syndrome of desirable psychological traits, typically seen in the perspective of psychologists and educators. Wikipedia defines fuzzy concepts as notions of “which the content, value, or boundaries of application can vary according to context or conditions, instead of being fixed.” Another name for this kind of ideas is “sensitizing” concepts as opposed to “definitive” concepts. Though positive psychologists tend to refer to similar characteristics, there is no agreement about the precise mix of these characteristics.

The use of the term “health” suggests that these characteristics should be part of our natural repertoire and are not a cultured exception, a suggestion that is difficult to support as we will discuss in “Assumed functionality of the traits deemed ‘healthy’.” What present day positive psychologist call “health” was referred to as “virtue” by their predecessors in educational and character-building professions.

The variety in notions of positive mental health prevalent today is illustrated in the following examples.

In her seminal exploration of this notion Jahoda (1958) listed the following six elements of positive mental health:

- Positive attitudes toward the self
- Growth, development, and self-actualization, including utilization of abilities, future orientation, and concern with work
- Integration, as in a balance of psychic forces, the unifying of one’s outlook, and resistance to stress and frustration
- Autonomy, as in self-determination, independent behavior, and, when appropriate, non-conformity
- A true perception of reality
- Environmental mastery, meaning adequacy in love, work and play, adaptation and adjustment, and the capacity to solve problems

Additional strengths mentioned by Keyes (2005) are:

- Positive relations with others having warm, satisfying, trusting personal relationships, and being capable of empathy and intimacy
- Purpose in life holding goals and beliefs that affirm one’s sense of direction

From the perspective of self-determination theory Deci and Ryan (2002), the following *motivations* are seen as part of positive mental health

- Autonomy (needing to be self-regulating; to own one’s actions and to identify one’s self with one’s behavior)
- Competence (needing to be effective; to be moving toward greater mastery and skill)
- Relatedness (needing to feel psychological connection with important others; to support, and be supported by, those others)

The British Mental Health Foundation (2021) stresses the following *abilities*:

- Ability to learn
- Ability to feel, express, and manage a range of positive and negative emotions
- Ability to form and maintain good relationships with others
- Ability to cope with and manage change and uncertainty

A closer look at the various descriptions of positive mental health by Peterson and Seligman (2004) reveals that the notion does not only include multiple mental strengths, but also external conditions that facilitate well-functioning (enablers) and results of well-functioning (outcomes).

Given this multitude of meanings, it is difficult to understand what PPIs improve. This is less problematic when life-satisfaction is used as an outcome measure, at least when it is defined as “the overall appreciation of one’s life-as-a-whole (Veenhoven, 2019).”

## Incomparable Measures of Positive Mental Health

This difference in the conceptualization of positive mental health is mirrored in the ways positive mental health is measured. There is not one measure of positive mental health, but there are a number of different inventories, which contain varying sets of psychological strong points. A review of current scales can be found in Health Scotland (2007).

One problem with this practice is that many of the effect studies are incomparable. As different outcome measures are used, we cannot assess which interventions work best for what kinds of people.

Another problem is that the sum-scores obtained using these inventories do not give us information about what precisely has been strengthened by the intervention. Together this makes it difficult to assess what different PPIs really do, in spite of the statistical power of some advanced meta-analyses.

This is less of a problem for life-satisfaction. Life-satisfaction is a clear concept: how much you like the life you live. So, when life-satisfaction is used as an outcome measure it denotes the degree to which a PPI has added to the enjoyment of life of the client. This is not only clearer; but it is also what users of positive psychological trainings typically want to achieve in the end.

## Objective Mental Health is Not Well Measurable Using Subjective Self-Estimates

Another problem is in the technique used to measure positive mental health, which is typically done using self-report questionnaires, such as the “Mental Health Continuum” (DeLare-Machido and Ruschel-Bandeira, 2015) and the “Positive Mental Health Scale” (Lukat et al., 2016).

Conceptually, positive mental health denotes several objective phenomena, in Jahoda’s list given above; we see only one subjective element that is “positive attitudes toward the self.” The other five elements of positive mental health are all of an objective nature: “growth,” “integration,” “autonomy,” “true perception of reality,” and “environmental mastery.” These objective phenomena are not well measurable using self-ratings as we know from the case of intelligence, where self-rated intelligence appears to be only modestly correlated with tested IQ (Paulhus, 1998). The problem is not just in self-serving bias but mainly because we do not know to judge our intelligence for ourselves. The same holds for our self judgements of growth, psychological integration, and reality control. This problem was already addressed in 1958 by Marie Jahoda but is being ignored in present day research practice in positive psychology.

This problem does not exist for self-determining life-satisfaction, which is a subjective phenomenon by definition, and can be easily and validly measured using self-reports (Veenhoven, 2019).

## Assumed Functionality of the Traits Deemed “Healthy”

Why are the psychological traits discussed above in “Different notions of positive mental health” seen as “healthy”? A main reason is in the functionality of such traits in the context of contemporary modern society. Although the functionality of traits such as a “true perception of reality” and “environmental mastery” is likely to be universal, having “autonomy” and “positive self-regard” will be more advantageous in modern individualized societies than owning the same traits in traditional collectivist societies. Modern multiple-choice societies provide many options for leading one’s life, among other things in the realm of choosing an occupation. Making a choice that fits one’s capabilities and preferences requires that one know oneself and that one has the guts to resist pressures toward being pushed into a less well-fitting way of life for yourself, such as following the footsteps of your parents into a family business. Autonomy is less functional in a collectivist context, where “relatedness” will be more important. Taking a historical view, we must realize that bravery and loyalty were extremely functional for the medieval warrior caste and were, as such, cultivated by the educators, trainers, and moral advisors of that time.

Although the strengths considered to mark positive mental health tend to be functional in contemporary modern society, they are not equally functional in all conditions. The benefits of self-determination will depend on the availability of options and the realistic perception of these. Also, more self-determination is not always better; one can be too independent, even in a modern individualized society we need support and corrections from our fellow men. The commonly used “forgiveness” interventions (e.g., Schulz, 2021) helps to illustrate the above points: one can doubt that it is helpful for a Holocaust survivor to write a forgiveness letter to Adolf Hitler and if the Holocaust survivor gets any better from forgiving Adolf Hitler at all, even a moderate degree of forgiving is probably better than forgive him completely.

Given the above, the effect of PPIs should not only be measured by the degree to which the trained strengths are strengthened, but also how this works out in a person’s adaptation to life after a PPI. This is where life-satisfaction comes in. Satisfaction with life reflects the degree to which one’s needs and wants are being met (Veenhoven, 2020). As such, life-satisfaction indicates an *actual outcome* in the quality of a life rather than a gain in *presumed means* (strengths) for living a good life.

## Poor Measures of Life-Satisfaction Used

Though the focus of current effect studies is on positive mental health, many studies also include measures of life-satisfaction. Commonly used measures are the “Satisfaction with Life Scale: SWLS” (Diener et al., 1985) and the “Subjective Happiness Scale: SHS” (Lyubomirsky and Lepper, 1999): both of these are multiple item questionnaires of which one of the questions does not fit a strict definition of life-satisfaction.

In the case of the five-item SWLS that is the endorsement required for the statement “If I could live my life over again, I would change nothing.” Logically, one can be satisfied with one’s life, but still be open for something else. The item is particularly inapt for measuring the effect of happiness training techniques, since users of such techniques are typically seeking to change their lives.

In the case of the four-item SHS, the problem lies in the statement “Compared to other people, I consider myself less happy/happier.” Logically, one can think one might be happier than other people, but still be unhappy. Practically, we are often badly informed about how happy “other” people are. This item is also particularly inapt in the context of an effect study. We tend to think that we are happier than other people anyway (Klar and Giladi, 1999) and participation in a PP training is likely to foster this illusion.

In my view, such lacks of substantive face-validity cannot be offset by mathematical “tests” for concurrent validity or construct validity. Gathering research findings on effects of happiness trainings on happiness, Bergsma et al. (2020) had to bypass more half of the available research output for this reason. These seemingly mere technical problems are part of a tradition in psychological measurement that is fitted to the fuzzy concepts, which prevail in the discipline. If you cannot define a concept precisely, you can only measure it using approximations, and such guestimates are inherently indefinite.

This is not a problem when life-satisfaction is measured using one or more questions that clearly address this clear concept, such as the question “Taking all together, how satisfied or dissatisfied are you with your life-as-a-whole these days?” When life-satisfaction is used as an outcome variable, we therefore assess whether the questions we use to assess it precisely fit this concept. This can be assessed on the basis of close reading of questions. Measures that have passed the test for face validity are listed in the Collection of Happiness Measures of the World Database of Happiness (Veenhoven, 2021). Their applicability for specific purposes is discussed in Veenhoven (2017).

In theory, studies in which poor measures of life-satisfaction have been used can be re-analyzed, using scores from which any unsuitable items have been removed. In practice, this appears to be difficult as I have experienced from making several requests to investigators; the original investigator is often no longer available or, if contactable, unwilling.

## Reservations About Life-Satisfaction in Positive Psychology

The prime aim of positive psychologists is to cultivate psychological virtues and as such most of them see happiness, at best, as one of these virtues or as a secondary byproduct. In this context, a distinction is made between “eudaimonic” happiness (a fancy name for positive mental health) and “hedonic happiness” (life-satisfaction), where the latter is often denounced as superficial pleasure and associated with hedonism. See for the example, the statement of Seligman (2002) on the Pleasant Life as “consisting in having as many pleasures as possible and having the skills to amplify the pleasures.”

Although pleasure will typically contribute to life-satisfaction, it should not be equated with life-satisfaction. One can indulge in pleasures but still be dissatisfied with one’s life as a whole, and one can be satisfied with life without experiencing much pleasures, for instance, when satisfaction draws primarily on perceived meaning. This conceptual difference is explained in more detail in Veenhoven (2020). Likewise, life-satisfaction should not be equated with hedonism. Although pleasure seeking and acceptance of pleasure will typically add to life-satisfaction (Veenhoven, 2003), they are not the same.

This is not to say that the use of life-satisfaction as an outcome variable is problem free. The main goal of PPIs is typically not that people should *feel* better but that they *do* better in life. Though life-satisfaction is a good indicator of actual adaptation (Veenhoven, 2020), it is not a perfect measure. Cognitively, one can be satisfied with a poor life because or ignorance or defensive denial and such mis-judgement will be corrected only by affective experience when gratification of basic needs is thwarted. Likewise, we can feel bad most of the time (affective component of life satisfaction) while there is nothing wrong, as is the case with common affective disorders such as depression.

Though imperfect, life-satisfaction is still the best available measure of adaptation to life. It is affective component in particular is both a signal of good adaptation that exists in all choice making organisms and at the same time a facilitator of active adaptation (Fredrickson, 2004). Progress in research on the effects of PPIs requires that the qualms about life-satisfaction are overcome.

The reservations about using life-satisfaction for measuring the effect of PPIs may also have a practical ground, small interventions being unlikely to have a demonstrable effect on satisfaction with life-as-a-whole, in particular long-term effects. If so, the effectiveness of PPIs can only be demonstrated by looking for the improvement of the traits trained in the short-term. Yet this is not what we want and, in my view, is not the only option.

There is evidence of effects of PPIs on life-satisfaction, such as in the case of “happiness training,” which tends to raise life-satisfaction by some 5% (Bergsma et al., 2020). Although the effects of PPIs may less well reflect in responses to questions on overall life-satisfaction, they are more likely to manifest in measures of the affective component of life satisfaction, such as measured with affect balance scales, which typically assess mood over the last few weeks. This difference between “overall” life-satisfaction and its affective and cognitive “components” is explained in Veenhoven (2009).

Anyway, we must acknowledge that PPIs are not designed for a short-term fix, but to foster traits that benefit a client in the long-term. Thus, it is worth knowing, which PPIs do this for what kinds of people.

## Conclusion

Effect studies on PPIs should use life-satisfaction as an outcome variable because: (1) Life-satisfaction is a clear concept, and as such denotes an identifiable effect; (2) Life-satisfaction can be effectively measured using self-reports, which is not the case for objective notions of positive mental health; (3) Life-satisfaction can be compared across studies, which is required to get a perspective on what works for whom; and (4) Life-satisfaction signals *actual* adaptation to life rather than presumed *chances* for adaptation; it therefore denotes an *end* result that the users of PPIs typically seek, rather than an improvement in the inner *resources* that may benefit them.

## Data Availability Statement

Publicly available datasets were analyzed in this study. This data can be found at: World Database of Happiness: https://worlddatabaseofhappiness.eur.nl.

## Author Contributions

The author confirms being the sole contributor of this work and has approved it for publication.

## Conflict of Interest

The author declares that the research was conducted in the absence of any commercial or financial relationships that could be construed as a potential conflict of interest.

## Publisher’s Note

All claims expressed in this article are solely those of the authors and do not necessarily represent those of their affiliated organizations, or those of the publisher, the editors and the reviewers. Any product that may be evaluated in this article, or claim that may be made by its manufacturer, is not guaranteed or endorsed by the publisher.
